# Chicago Medical Society

**Published:** 1885-07

**Authors:** 


CHICAGO MEDICAL SOCIETY.

*Stated Meeting, July* 6, 1885.—The First Vice-President, C. W. PURDY, M. D., in the chair.

DR. ROBERT TILLEY exhibited to the society microscopical specimens of the fungus, aspergillus glaucus, taken from the human ear, an osteoma developed from the crusta petrosa of a canine tooth, and filaments, or mycelia, from the body of a tonsil.

While exhibiting the specimens, DR. TILLEY spoke as follows:

In describing to you the three specimens which are exhibited under the microscope, I will refer in the first place to the fungus—aspergillus glaucus—taken from the human ear. I have had this in my possession for about three years and have shown it to several of my acquaintances, but did not deem it of sufficient interest to exhibit it before. It is however an object which many have not seen before, although it is mentioned in every text-book on the subject I have no intention whatever of entering into the question of the aspergilli in general relative to their influence when found in the ear. But I must say that my experience relative to the question leads me to think that its influence as a source of pain in the external ear is greatly exaggerated in the books. This specimen was taken from the posterior wall of the meatus of a little girl who had for sometime previously been afflicted with otorrhœa. The otorrhœa had however ceased and the fungi were readily recognized in lusty growth immediately on looking into the ear. There was however no pain complained of, the patient was brought rather for inspection than for the expectation of relief. You will observe that the fungi are growing on what by simple inspection might be called dried but otherwise normal wax. You will notice that the fungus consists of one straight long stem surmounted by a round ball, very much like the top of an onion which has run to seed. It is commonly said, I think rather on theory than on observation, that they are caused by sleeping in low, damp apartments. The child from whom this was taken belonged to people in good circumstances and was well taken care of and was not living in damp quarters.

The next specimen, to which I will now refer, is the osteoma developed from the cementum or crusta petrosa of a canine tooth. I am very sorry that I cannot give you anything of the clinical history, because I believe it would be interesting, if known. In consequence of this, it is perhaps necessary that I should give a word of explanation as to how it came into my hands :—A friend was speaking to me of some one who had been subjected to the operation of drilling through the fangs of six teeth on account of what was called " ossification of the nerve. " In speaking to one of my acquaintances among the dentists about such a condition, I was presented with a tooth a section of which I exhibit to you. You will see, both macroscopically as well as microscopically, that the line of demarcation is well defined. You will further see that the general appearance of the tumor is that of bone, and that it differs greatly from the general appearance of the tooth proper. It is interesting to observe moreover that the canal, through which the nerve and vessels enter, is greatly diminished in its course through the tumor, consequently great pressure must have been exerted on the nerve. Before making the section, I had supposed that the canal was completely obliterated, so small is its opening at the end of the tumor. On looking at the specimen through the microscope, you will see very clearly that while the line of demarcation between the dentine and the proliferation of the crusta petrosa is well marked, and the lacunæ and canaliculæ of the bony structure of the tumor are well demonstrated, there is also a number of contorted tubules in the bony tumor which resemble, somewhat, the dental tubules. Haversian canals are of course not present, they never are in such growths. Although I have no clinical history to present, I may add that the usual clinical history is one of severe pain, which nothing but extraction seems capable of relieving.

The last specimen is one which I obtained from one of those little pockets which are often found in the tonsils. They seem to come and go, sometimes without giving any more inconvenience than a little discomfort. They are frequently associated with fœtid breath, and in some cases the masses themselves are very offensive, in odor. In the present case, however, this was not so, there was no fœtor. There was, however, an unpleasant sensation amounting to a positive discomfort running down the neck externally, in the direction of the sterno-cleido-mastoid muscle. On pressure around the base, the little mass popped out, suddenly, so that it came near going down the patients throat. In examining it, under the microscope, it proved to be one mass of filaments, very fine and containing spores in the body of the filaments, and associated with fat crystals.

I succeeded in staining them with methyl violet, but only after first extracting the fat with ether.

I have no theory to present, gentlemen, I simply exhibit to you what has been interesting to me. After the removal of the small mass I did not think any treatment was necessary, but as there was a little bridge of tissue more or less dividing the cavity into two sections, I divided the bridge with the electro-cautery.

*The Treatment of Acute Coryza*, was the subject of a paper by DR. J. A. ROBINSON. He said the literature on the subject of the treatment of acute coryza is scanty and of a stereotyped nature. The profession seems to have arrived at two conclusions, first, that it is not a disease of sufficient severity and importance to command especial attention; second, that no plan of abortive or curative treatment has been sufficiently successful to cause them to investigate the subject further. However, in view of the fact that repeated attacks of acute coryza undoubtedly have a causal relation to pathological changes in the nares which it is difficult to remove, and that we are so frequently consulted by public speakers and singers who beseech us to abort or rapidly cure such acute attacks, it certainly deserves more than a passing notice.

The time-honored plan of aborting an acute coryza by the administration of a full dose of opium, an active purge and a potent diaphoretic has proven more disagreeable than efficacious. The plan, advocated by Dr. Ferrier, of blowing into the anterior nares a powder composed of morphia, bismuth and acacia, has been quite satisfactory in a few instances, but it is not free from the objection that, when successful, it often produces an unpleasant nausea. Its success is undoubtedly due to the sedative and astringent effect upon the inflamed mucous membrane.

What are the pathological conditions in the first stage of acute coryza? Briefly, there is dilatation of the capillary vessels, the arterioles being dilated and the venules engorged, inducing tumefaction of the mucuous membrane. This is accompanied by dryness and pain. Secretion is abolished. In reflecting upon these circumstance the thought naturally arises, whether, if we can employ such measures or drugs as will antagonize these abnormal states, we will succeed in aborting the disease. We have recently had added to our armamentarium a drug which more completely antagonizes in its physiological actions these pathological conditions than any other. It is the hydrochlorate of cocaine.

Its physiological actions have been demonstrated to be concisely as follows: when applied to a mucous membrane it is a potent although transient anæsthetic, a vaso-motor constrictor causing contraction of the arterioles and depletion of the venules, thus rapidly emptying congested tissues of a surplus of blood. This drug is also an astringent and has the property of lessening the secretion of muciparous glands. On studying the relation between the state of the nasal mucous membrane in the first stage of acute inflammation and after an application of cocaine, the theory was formulated that cocaine should prove useful in aborting acute coryza, and it was determined to try it on the first opportunity.

The details of the first experiment are as follows:

Miss S———, a soprano singer in one of our city churches, applied to me on the morning of February 22nd, and desired immediate relief from a "cold in the head." She complained that the previous night she had been exposed to a draft and awoke that morning with the cold, as evidenced by the fact that she could not breath through the nose, and that her nose felt dry and painful, and she had lost the sense of smell. Inasmuch as she had to sing that night at a special service, she must have immediate relief.

Upon examination I found all the conditions incident to the incipiency of an acute coryza. Her temperature was 102° F. with some acceleration of the pulse.

Febrifuges and a mild diaphoretic were prescribed. A local application of a four per cent. solution of hydrochlorate of cocaine was applied, as thoroughly as possible, to the congested mucous membrane, and the parts were sprayed, also, for some time with a warm alkaline spray, hoping thereby to reduce the hyperæmia. After having made another application of the cocaine, the patient was instructed to return home and follow the same line of treatment and to return the following day. She did not return until three days later, when she reported, to my surprise and gratification, that she had been able to sing as desired, and that no symptoms of the disease had returned.

The success which attended this new departure, induced me to try it in other cases of acute coryza which were seen early, and it has almost always been successful. Of course, the number of cases which we see in their forming stage are few, on account of the fact that the patients do not seek medical advice for this affection until the disease is well advanced.

In the use of cocaine for the purpose of aborting an acute coryza there are some objections: it has to be applied often in order to maintain its action on the inflamed mucous membrane, and it is an expensive drug. I have found that the use of a warm alkaline spray serves to prolong the sedative action of the cocaine.

Of course dependence is not to be placed on local measures alone, but in addition proper attention is to be given to constitutional and hygenic treatment.

Dr. Tilley said he had used the hydrochlorate of cocaine in two or three cases of acute coryza with much satisfaction. According to one patient, an attack had ended with a single application. While he did not look upon cocaine as a sure cure for acute coryza, he thought it almost always did good. He referred to a serious accident which occured to one of his patients during the use of cocaine. The patient was a boy aged twelve years, in whose nose a little cocaine had been used. After the first application he suffered a little nausea, which was not regarded as serious; after the second application the nausea was worse, but it was not until a third application had been made that the symptoms became alarming. These symptoms were difficulty of breathing, syncope, irregular action of the heart, cold perspiration and loss of sensation in the extremities. Notwithstanding these symptoms were alarming, the boy recovered quite rapidly. He had noticed reports of cases in the journals where the same symptoms had appeared.

Dr. Weller said that he had had a good deal of experience in the use of coca, especially in the form of the fluid extract. He had taken large doses, in his own case he had used two pounds in a short time. Formerly he had considered it as harmless as tea, but latterly he had arrived at the conclusion that it is a powerful narcotic. The strange phenomena which follow use of cocaine in some cases, he believes to be due to the narcotic action of the drug, and that they would not appear if the drug was not given in large doses. He believed some patients to be peculiarly susceptible to the action of coca or cocaine, similar to the idiosyncrasies of patients in the use of belladonna, opium and alcohol. In the case mentioned, he believed the symptoms to have been the result of an over dose of cocaine. In his experience, he had found a two per cent. solution of cocaine strong enough, and urged the tentative use of the drug in the same manner as in the use of morphia.

Dr. WEBSTER did not wish to be considered skeptical, but he had some doubt as to the alarming symptoms in the cases mentioned having been due to the drug. Is it not possible they were the result of reflex processes in over-sensitive patients? He had a patient recently who vomited after holding a fever thermometer under her tongue.

Dr. PAOLI believed that the old treatment of acute coryza by giving the patient a hot bath, muriate of ammonia internally, and inhalations of camphor in hot water, or the oil of eucalpytus, combined with borax, to be the best, although he would acknowledge that cocaine would often relieve severe attacks in a short time.

Dr. S. J. JONES asked the author of the paper if he had used cocaine with a steam atomizer in acute pharyngitis, tonsillitis and laryngitis; also, if the applications of cocaine to different patients were from the same solutions and at brief intervals, so as to be able to state how a reliable solution acted on different patients.

Dr. Robison, in closing the discussion, said he did not advocate the plan of treatment as infallible or free from objections, nor did he neglect to use other means of cure if he thought they were advisable. As to the effect of cocaine on certain patients, he had a similar experience to Dr. Tilley in the case of a woman who had twice been operated on without cocaine for nasal polyps. No unfavorable symptoms occurred during these operations. At the third and fourth operations, cocaine was used and the patient was troubled with nausea, vomiting, palpitation of the heart and syncope. As no cocaine had been used in the first two operations, these symptoms in the third and fourth operations seemed to be, undoubtedly, due to an idiosyncrasy of the patient. He had not used cocaine with the steam atomizer, but he thought it feasible, if the drug were not so expensive. He prepares fresh solutions for each patient so as to preclude all possibility of failure of action by reason of deterioration of the solution by age. He had found the same package of cocaine to vary in its local and constitutional effect on different patients, affecting some more rapidly and profoundly than others.

Society then adjourned.

